# Case report: Urachal perivascular epithelioid cell tumor

**DOI:** 10.3389/fonc.2024.1324193

**Published:** 2024-03-26

**Authors:** Mengru Liu, Pan Liang, Dongbo Lyu, Bingbing Zhu, Jianbo Gao

**Affiliations:** Department of Radiology, The First Affiliated Hospital of Zhengzhou University, Zhengzhou, China

**Keywords:** urachal tumor, urachus, PEComa, imaging features, case report

## Abstract

**Background:**

Urachal tumors are rare in clinical practice, among which urachal adenocarcinoma is the most common. In this study, we report a rare case of urachal perivascular epithelioid cell tumor to improve our understanding of the disease.

**Case presentation:**

A 26-year-old male patient was hospitalized for lower abdominal pain. The US showed a hypoechoic mass measuring 26mm × 18mm in the superior aspect of the bladder. MRI showed an irregular mass located anterior to the bladder roof, near the midline. The tumor exhibited hypointense on T1WI and heterogeneous hyperintense on T2WI. Additionally, contrast-enhanced T1-weighted imaging revealed obvious ring enhancement of the tumor. The patient underwent surgical resection of the urachal tumor, with subsequent pathological examination revealing a diagnosis of urachal PEComa. Following surgery, the patient underwent regular follow-up assessments, with no evidence of recurrence or metastasis observed after three and a half years.

**Conclusions:**

Urachal PEComa is a rare mesenchymal tumor that presents challenges in diagnosis through imaging and clinical symptoms. Definitive diagnosis relies on pathological and immunohistochemical analysis. Due to the rarity of urachal PEComa, prognosis assessment necessitates long-term follow-up and evaluation of more cases.

## Introduction

The urachus is a remnant of the embryonic allantois, which is located in the loose connective tissue between the peritoneum and the transverse fascia. It is usually occluded before birth and during infancy into a non-functional fibrous cord known as the median umbilical ligament. Incomplete urachus degeneration can lead to cysts, diverticulum, tumors, and other lesions (1). Urachal perivascular epithelioid cell tumor (PEComa) is extremely rare. Here, We report a case as follows.

## Case presentation

**Figure 1:**
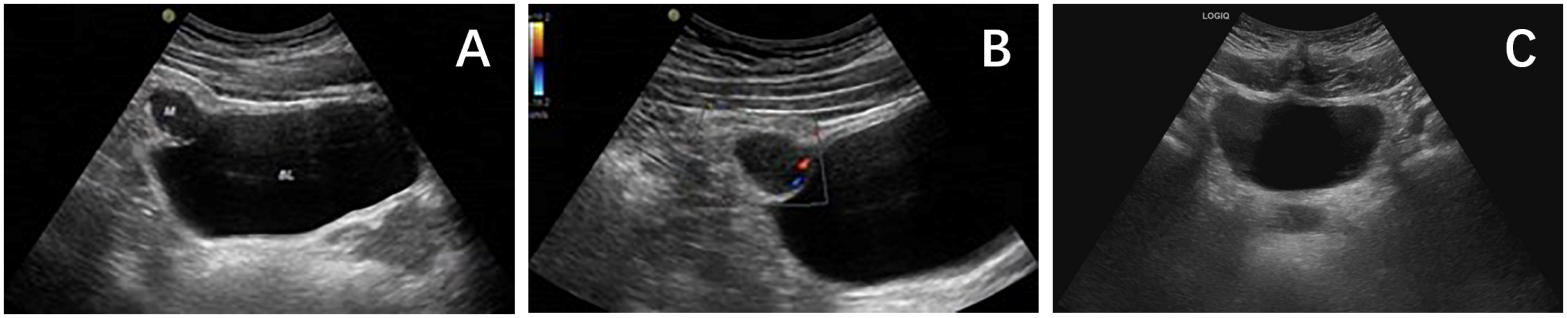
**(A)** Ultrasound showed that there was a hypoechoic mass of about 2.6×1.8cm in the upper part of the bladder. **(B)** CDFI showed dot linear blood flow signals at the edge of the mass. **(C)** Ultrasound showed no obvious abnormalities in the bladder.

**Figure 2:**
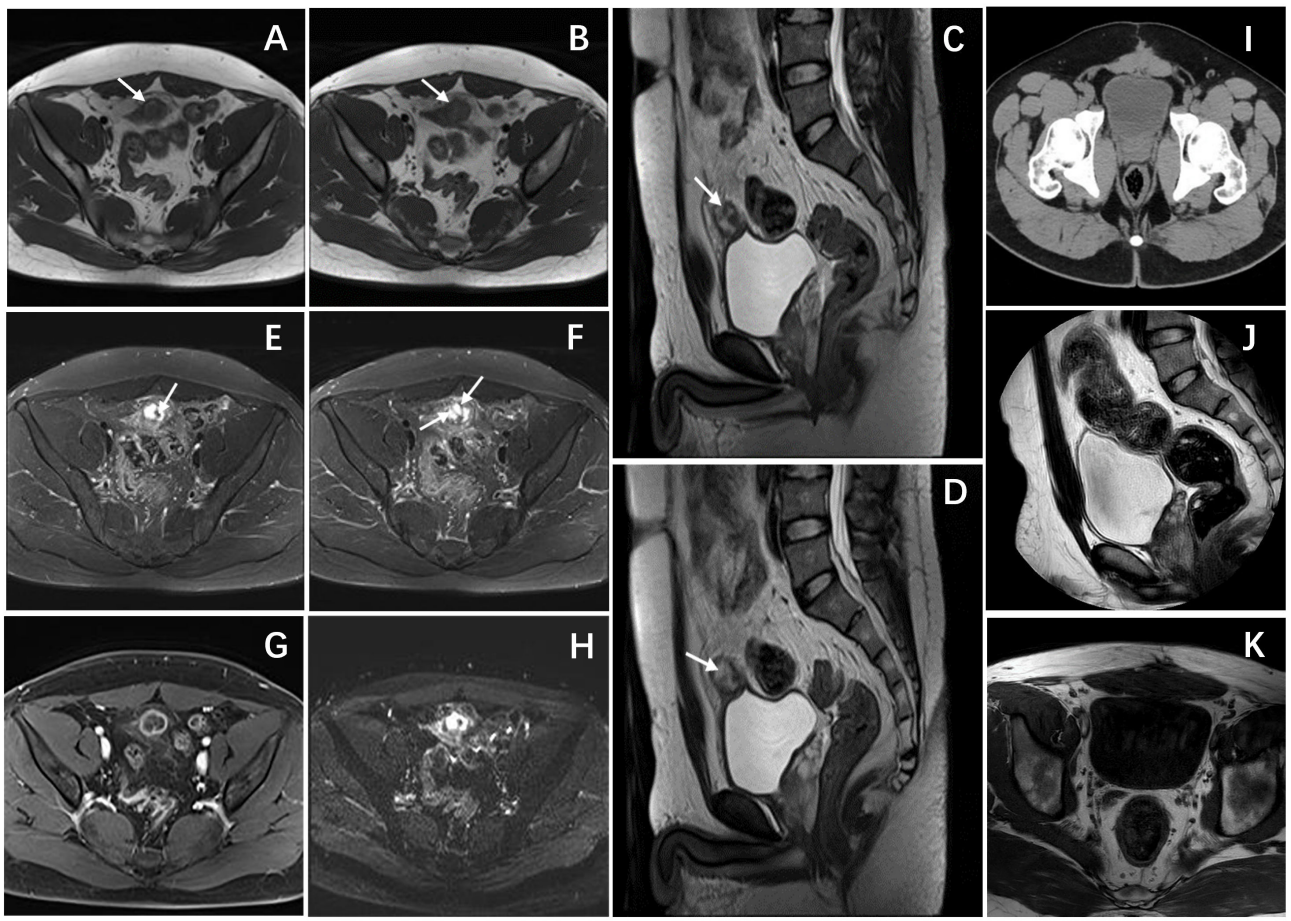
Pelvic magnetic resonance and CT images. **(A, B)** Axial T1-weighted images showed a hypointense mass. **(C, D)** Sagittal T2-weighted images demonstrated a heterogeneously hyperintense mass anterior to the bladder roof, with spot-line hypointense (arrows) in the mass. **(E, F)** Axial T2-weighted images with fat suppression showed similar imaging findings to T2WI, the mass still showing heterogeneously hyperintense with linear and punctate hypointense (arrows). **(G)** Axial T1-weighted enhanced image showed obvious ring enhancement of the mass. **(H)** Axial DWI showed limited diffusion of the mass. **(I)** An Axial CT scan showed that there was no thickening or mass shadow of the bladder wall. sagittal T2-weighted image **(J)** and Axial T1-weighted images **(K)** showed that the bladder was full, and there was no obvious abnormal signal in the bladder cavity.

**Figure 3:**
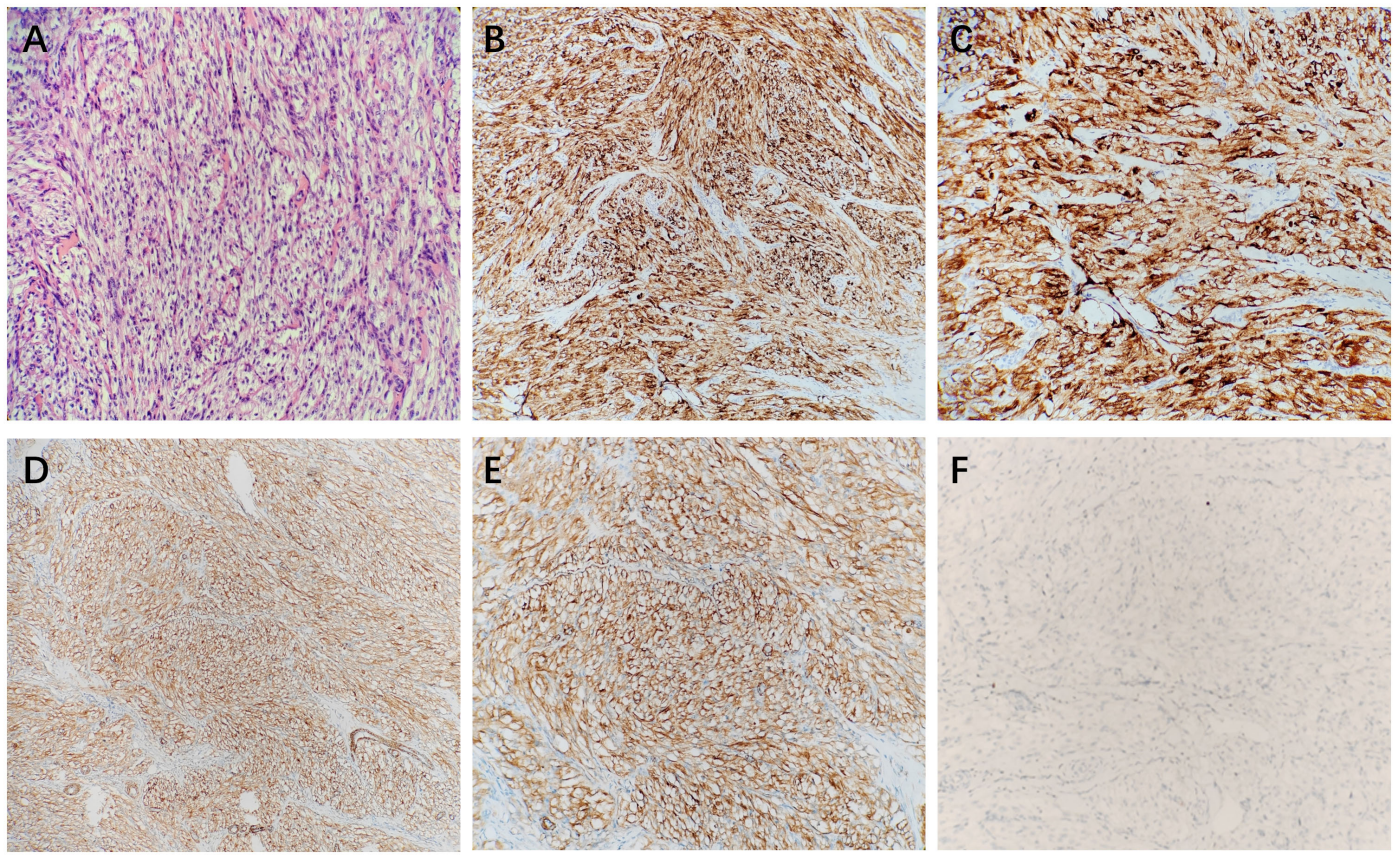
The microscopic view showed that the tumor cells were spindle-shaped **(A)**. Immunohistochemical stains showed that HMB45 **(B, C)**, and SMA **(D, E)** were positive, and Melan-A **(F)** was negative.

A 26-year-old male patient sought medical attention at a local hospital for lower abdominal pain, which was found to be associated with a hypoechoic mass located in the anterior superior region of the bladder upon ultrasound (US) examination. Subsequently, the patient was referred to our institution for further evaluation and management, where he was admitted for further assessment. Following admission, specialist physical examination and laboratory investigations did not reveal any significant abnormalities. Cystoscopy examination demonstrated no apparent abnormalities within the bladder cavity. The US revealed the presence of a hypoechoic mass measuring approximately 26mm × 18mm in the upper aspect of the bladder (**Figure 1A**). The mass exhibited a distinct boundary, irregular shape, and uneven internal echo. Color Doppler flow imaging (CDFI) revealed dot linear blood flow signals at the edge of the mass (**Figure 1B**), supporting the diagnosis of an urachal mass. Magnetic resonance imaging (MRI) depicted an irregular mass located anterior to the bladder roof near the midline (**Figures 2A–H**), measuring approximately 24 mm × 24 mm × 25 mm in dimensions (left and right diameter × anteroposterior diameter × upper and lower diameter). The tumor exhibited hypointense on T1-weighted imaging (T1WI), heterogeneous hyperintense on T2-weighted imaging (T2WI) and fat-suppression imaging, and hyperintense on diffusion-weighted imaging (DWI). The demarcation between the lesion and the dome of the bladder was indistinct, with patchy hyperintense observed in the adjacent soft tissue on fat-suppression imaging. The tumor displayed obvious ring enhancement on contrast-enhanced T1-weighted imaging. Given the high likelihood of infection, a diagnosis of urachal abscess was made. Then the patient underwent urachal tumor resection. During the surgical procedure, a round extraperitoneal mass measuring approximately 3 cm × 2 cm was identified. The upper aspect of the mass was situated 2 cm below the umbilicus, with the lower aspect connected to the bladder dome. Gross examination revealed a tumor measuring 3.5 cm × 2.8 cm × 2.0 cm, characterized by a distinct border and moderate firmness. The cut surface exhibited a gray-yellow or gray-brown appearance. Microscopic analysis revealed spindle-shaped tumor cells (**Figure 3A**) displaying infiltrative growth patterns with areas of necrosis. The tumor cells exhibited high nuclear grade and a significant number of mitotic figures. The immunohistochemical staining results indicated negative expression of AE1/AE3, EMA, S-100, desmin, ALK, SOX-10, caldesmon, and Melan-A (**Figure 3F**), while positive expression was observed for SMA (**Figures 3D, E**), calponin, CD34, HMB45 (**Figures 3B, C**), and Ki-67, with a Ki-67 positive rate of approximately 5%. The final diagnosis of malignant urachal PEComa was made. The patient had no significant postoperative discomfort and was discharged. Regular follow-up examinations over a period of three and a half years revealed no recurrence or metastasis (**Figures 1C**, **2I–K**).

## Discussion

### Clinical features

PEComa is a mesenchymal tumor composed of histologically and immunohistochemically distinct perivascular epithelioid cells (2). The concept of PEComa was first proposed by Bonetti et al. in 1992 (3). The PEComa family includes various tumors such as angiomyolipoma, lymphangioleiomyomatosis, clear cell “sugar” tumor of the lung, primary extrapulmonary “sugar” tumor, clear cell myomelanocytic tumor of the falciform/round ligament, and other tumors with similar features in different anatomical locations (4).

The urachus is a 5-10cm tubular structure extending from the umbilicus to the dome of the bladder. When the structure is not fully degenerated, a variety of urachal lesions can be formed, most commonly including the following four types: ①urachal cyst, the urachal ends are closed, but the middle lumen is not closed; ②urachal sinus, the umbilical part of urachal is not closed, but the bladder end is closed; ③urachal fistula, the urachal is completely unclosed, and the umbilical part is communicated with the bladder; ④urachal diverticulum, the umbilical part of urachal is closed, but the proximal end of bladder is not closed. Among the various types of urachal lesions in adults, urachal cyst is the most prevalent, representing over 50% of urachal abnormalities. It is usually found incidentally. When the cyst is secondarily infected, symptoms such as abdominal pain, lower abdominal mass, and fever may occur (1, 5). Furthermore, incomplete urachal regression can lead to the development of urachal tumors. Urachal tumors are rare in the clinic, accounting for about 0.35%-0.7% of bladder tumors, of which about 90% is urachal adenocarcinoma (6). Urachal PEComa is a rare neoplasm with an unknown incidence rate.

As with PEComa in other sites, PEComa of the urinary system also occurs in young and middle-aged patients. However, unlike PEComa of soft tissue, skin, and bone, PEComa of the urinary system is more common in men (man: woman = 2:1), while PEComa of other sites is more common in women (man: woman = 1:7) (7, 8). Previous research suggests that patients with urinary system PEComa often present with nonspecific symptoms, with small, asymptomatic tumors frequently being discovered incidentally. Patients with bladder PEComa may present with abdominal pain, hematuria, dysuria, and other symptoms (9). Patients with renal or ureteral PEComa may present with lumbago, hematuria, fever, and other symptoms (10, 11). Due to the special location of the urachal, urachal tumors often have an insidious onset, and most patients are diagnosed with hematuria, lower abdominal pain, and abdominal mass (5, 6). The patient with urachal PEComa in our study was a young man who presented with lower abdominal pain as the main symptom.

### Histologic and pathologic features

PEComa is a rare neoplasm that can occur in various anatomical locations. Predominant sites of occurrence include the kidney and uterus, with documented cases also observed in the bladder, ureter, liver, lung, gastrointestinal tract, and parapharyngeal region (12). Histologically, PEComa is characterized by epithelioid cells forming sheets and nests surrounding blood vessels, exhibiting clear or eosinophilic cytoplasm. Some tumor cells are spindle-shaped (7, 13). The distinctive immunoreactivity of PEComa is the expression of melanocyte-derived markers and muscle-derived markers. Almost all PEComa demonstrate positivity for HMB-45 and/or Melan-A, with a high proportion also expressing SMA and desmin (2, 14). In the case of urachal PEComa examined in this study, spindle-shaped tumor cells were identified microscopically, with positive immunohistochemical staining for SMA and HMB45 consistent with previous literature.

Most PEComas are benign, but malignant tumors have also been reported. Folpe et al. considered that PEComa can be classified as “benign”, “uncertain malignant potential” and “malignant”, and proposed the classification criteria of benign and malignant PEComa: (1) tumor diameter ≥ 5 cm; (2) The tumor shows invasive growth; (3) The tumor cells have high nuclear grade; (4) mitotic count ≥ 1/50 HPF; (5) necrosis of tumor cells; (6) vascular invasion. If the tumor does not have any of the above relevant features, it is considered “benign”; if there is one of the above characteristics, it is considered “uncertain malignant potential”; and if two or more of these related characteristics are present, “malignant” is considered (15). In our case, the demarcation between the tumor and the bladder wall was indistinct, with pathological analysis revealing invasive proliferation and necrosis within the tumor. The tumor cells displayed a high nuclear grade and a significant number of mitotic figures, leading to the classification of the lesion as a malignant urachal PEComa.

### Imaging findings

Computed tomography (CT), MRI, and US are commonly used to detect urinary tract tumors. The imaging characteristics of PEComa in the urinary system lack specificity. CT demonstrates PEComa as a circular low-density mass with either homogeneous or heterogeneous enhancement on contrast-enhanced scans, with some tumors exhibiting distinct circular enhancement (4, 7, 12). MRI signal intensity of PEComa varies, with Zheng et al. reporting ureteral PEComa displaying a slightly heterogeneous high signal on T1WI, consistent with a heterogeneous high signal on fat-suppressed sequences, low signal on T2WI, and obvious enhancement on contrast-enhanced T1-weighted imaging (10). Fang et al. observed that renal PEComa exhibited heterogeneous isointense on T1WI, with punctate hyperintensity within it, heterogeneous hyperintense on T2WI, and heterogeneous mild enhancement on T1WI enhanced scan (11). The hyperintense observed on T1WI was attributed to hemorrhagic or protein fluid within the tumor, which remained hyperintense on fat-suppression sequence. Notably, there is currently a lack of literature documenting the ultrasound manifestations of PEComa within the urinary system. In our study, the case of urachal PEComa exhibited a hypoechoic mass located superior to the bladder on ultrasound imaging, characterized by irregular internal echogenicity and punctate linear blood flow signals at the periphery. Additionally, the tumor demonstrated hypointense on T1WI, heterogeneous hyperintense on T2WI and fat-suppression sequence, and prominent ring enhancement following contrast administration. Furthermore, our study revealed that urachal PEComa exhibited hypointense on T1WI, with abnormal linear and punctate hyperintense signal present within the hypointense area. The abnormal signal displayed hypointense on T2WI and fat-suppression sequence. The enhancement pattern of the abnormal signal was comparable to that of the ring-like enhancement observed at the mass periphery, similar to the cyst wall and intracystic septa. Due to its rarity, it is difficult to diagnose PEComa by imaging at present, but we believe that ring enhancement and compartmental-like changes may be features of urachal PEComa.

### Treatment and prognosis

There exist variations in treatment modalities for various types of urachal lesions. i) Urachal cyst, the predominant form of urachal lesion in adults, necessitates surgical intervention upon diagnosis due to the potential for recurrence and malignant progression. The operation requires the removal of all urachus and diseased tissue from the umbilical cord to the bladder cuff. For urachal cysts with infection, there are two treatment options. The first treatment option is incision and drainage first, and then selective resection. The second treatment option is one-stage surgical resection with modified antibiotic therapy. Studies have shown that the first regimen is superior in reducing the risk of postoperative complications and shortening the average length of hospital stay (16, 17). ii) When diagnosing urachal fistula, the recommended course of action is radical treatment involving the removal and ligation of the urachal. During the resection process, it is crucial to ensure that the urachal is detached from the bladder wall and ligated in order to prevent the development of a diverticulum at the top of the bladder after incomplete resection (18, 19). iii) Some patients with shallow urachal sinus can heal spontaneously after debridement and dressing change, while those with deep urachal sinus often cannot be cured for a long time and still need surgical resection. The treatment principle is the same as that of urachal fistula. iv) Urachal diverticulum may remain asymptomatic and not require specific treatment, but surgical intervention is necessary in cases of infection or stone formation. v) The optimal treatment for urachal PEComa remains undefined. However, urachectomy and partial cystectomy are the preferred surgical approaches for urachal tumors. The chemotherapy regimen for urachal tumors lacks standardization, with cisplatin-based and 5-fluorouracil-based regimens commonly utilized in clinical practice (20). We believe that this may have some reference value for the treatment of urachal PEComa.

Currently, surgical resection is the first choice for the treatment of benign PEComa, while there is no standardized treatment approach for malignant PEComa. Previous studies suggests that chemotherapy and radiotherapy have limited efficacy in treating PEComa, with some studies indicating that molecular targeted therapy may hold promise as a potential treatment modality (4, 10). A review of previous reports on PEComa in other sites of the urinary system reveals that small bladder PEComa may be managed with transurethral resection of bladder tumor (TURBT) alone, with some patients subsequently undergoing partial cystectomy following TURBT. Partial cystectomy or radical cystectomy are commonly utilized for the treatment of large PEComa in the bladder (7, 12, 13). Similarly, partial nephrectomy or radical nephrectomy are the preferred surgical interventions for renal PEComa. Following surgery, some patients may receive chemotherapy, with a subset experiencing recurrence and metastasis during post-operative follow-up (21, 22). Notably, there have been limited reported cases of ureteral PEComa to date. Zheng et al. reported a TFE3-positive malignant ureteral PEComa patient who underwent right ureteral lesion resection and ureteral dissection anastomosis, but the patient had no follow-up after surgery and the prognosis was uncertain (10). Due to the rarity of urachal PEComa, determining optimal treatment strategies and predicting prognosis remains challenging. In our study, the patient with urachal PEComa underwent resection of the urachal lesion and did not receive targeted therapy or additional treatment postoperatively. Up to now, the patient has been followed up for three and a half years, and no tumor recurrence or metastasis has been found.

### Differential diagnosis

Given the infrequency of PEComa, a definitive standard for distinguishing urachal PEComa from bladder PEComa has yet to be established. Nevertheless, numerous researchers have endeavored to differentiate between bladder and urachal tumors, and have outlined diagnostic criteria for the identification of urachal tumors: (1) The tumor is located in the roof of the bladder; (2) Absence of cystitis cystica or cystitis glandularis; (3) Predominant invasion of the muscular or deeper tissues with a sharp demarcation between the tumor and the surface epithelium, which is free of glandular or polypoid proliferation; (4) Ramifications of tumor in the bladder wall with extension to the space of Retzius, anterior abdominal wall or umbilicus; (5) No evidence of primary tumors at other sites (23–25).

In our study, the assessment of the clinicians, pathologists, and radiologists, as well as the aforementioned criteria, were taken into account to determine whether the PEComa originated in the urachal or bladder. Firstly, multiple imaging examinations of this patient showed that the tumor located near the midline in front of the bladder roof, with the main mass located external to the bladder. Secondly, while the demarcation between the tumor and the bladder roof was indistinct, no evident anomalies were observed in the bladder mucosa during cystoscopy. Furthermore, intraoperative examination indicated that the tumor was suspended in the abdominal wall, with one end attached to the umbilical cord and the other end connected to the bladder. It is noteworthy that the majority of bladder tumors are typically located in the trigone region, with only a small percentage found in the roof of the bladder. Taking into account these findings, along with the results of the pathological analysis, the diagnosis in the final pathological report of this investigation was identified as urachal PEComa.

Due to its rarity, urachal PEComa can be easily misdiagnosed and must often be distinguished from the following diseases: ①Urachal cyst. Patients with simple urachal cysts typically present with no clinical symptoms, a clear cyst boundary, uniform thickness of the cyst wall, and uniform signal of the fluid within the cyst. Imaging typically reveal hypointense on T1WI, hyperintense on T2WI, and no enhancement on enhanced scans (1). ②Urachal carcinoma. Urachal carcinoma predominantly occurs in middle-aged and elderly male patients, with hematuria being the prevailing symptom. The typical presentation of urachal carcinoma is that of a cystic solid mass displaying complex echogenicity on ultrasound imaging. On MRI, the cystic component demonstrates a hypointense signal on T1WI and a hyperintense signal on T2WI, while the solid component exhibits an isointense or slightly hypointense signal on T1WI and a slightly hyperintense signal on T2WI. Following contrast enhancement, the solid component demonstrates significant enhancement, whereas the cystic component shows no enhancement. Additionally, certain tumors may exhibit calcifications in the form of dots, spots, strips, or arcs, which can be found either centrally or peripherally within the tumor (26).

## Conclusion

In conclusion, urachal PEComa is an extremely rare mesenchymal tumor that is currently difficult to diagnose by imaging. When encountering a tumor located anterior to the bladder dome, characteristic features include hypoechoic appearance on ultrasound, uneven internal echo, and linear blood flow signals at the edge. Additionally, the tumor typically appears hypointense on T1WI, heterogeneously hyperintense on T2WI and fat-suppression sequences, and demonstrates marked ring-enhancement and compartmental-like changes on contrast-enhanced scans, warranting consideration of urachal PEComa as a potential diagnosis. The diagnosis still depends on pathological and immunohistochemical results, and the prognosis still needs to be evaluated by long-term follow-up of more cases.

## Data availability statement

The original contributions presented in the study are included in the article/supplementary material. Further inquiries can be directed to the corresponding author.

## Ethics statement

The studies involving humans were approved by the Institutional Review Board of the first affiliated Hospital of Zhengzhou University. The studies were conducted in accordance with the local legislation and institutional requirements. The human samples used in this study were acquired from the hospital’s medical record system. Written informed consent for participation was not required from the participants or the participants’ legal guardians/next of kin in accordance with the national legislation and institutional requirements. Written informed consent was obtained from the participant/patient(s) for the publication of this case report.

## Author contributions

ML: Writing – original draft, Writing – review & editing. PL: Writing – review & editing. DL: Writing – review & editing. BZ: Writing – review & editing. JG: Writing – review & editing.
